# Single-Trial EEG Analysis Predicts Memory Retrieval and Reveals Source-Dependent Differences

**DOI:** 10.3389/fnhum.2018.00258

**Published:** 2018-07-10

**Authors:** Eunho Noh, Kueida Liao, Matthew V. Mollison, Tim Curran, Virginia R. de Sa

**Affiliations:** ^1^Department of Electrical and Computer Engineering, University of California, San Diego, San Diego, CA, United States; ^2^Department of Psychology and Neuroscience, University of Colorado Boulder, Boulder, CO, United States; ^3^Department of Cognitive Science, University of California, San Diego, San Diego, CA, United States

**Keywords:** EEG, memory retrieval, old/new effect, multi-variate analysis, prediction

## Abstract

We used pattern classifiers to extract features related to recognition memory retrieval from the temporal information in single-trial electroencephalography (EEG) data during attempted memory retrieval. Two-class classification was conducted on correctly remembered trials with accurate context (or source) judgments vs. correctly rejected trials. The average accuracy for datasets recorded in a single session was 61% while the average accuracy for datasets recorded in two separate sessions was 56%. To further understand the basis of the classifier’s performance, two other pattern classifiers were trained on different pairs of behavioral conditions. The first of these was designed to use information related to remembering the item and the second to use information related to remembering the contextual information (or source) about the item. Mollison and Curran (2012) had earlier shown that subjects’ familiarity judgments contributed to improved memory of spatial contextual information but not of extrinsic associated color information. These behavioral results were similarly reflected in the event-related potential (ERP) known as the FN400 (an early frontal effect relating to familiarity) which revealed differences between correct and incorrect context memories in the spatial but not color conditions. In our analyses we show that a classifier designed to distinguish between correct and incorrect context memories, more strongly involves early activity (400–500 ms) over the frontal channels for the location distinctions, than for the extrinsic color associations. In contrast, the classifier designed to classify memory for the item (without memory for the context), had more frontal channel involvement for the color associated experiments than for the spatial experiments. Taken together these results argue that location may be bound more tightly with the item than an extrinsic color association. The multivariate classification approach also showed that trial-by-trial variation in EEG corresponding to these ERP components were predictive of subjects’ behavioral responses. Additionally, the multivariate classification approach enabled analysis of error conditions that did not have sufficient trials for standard ERP analyses. These results suggested that false alarms were primarily attributable to item memory (as opposed to memory of associated context), as commonly predicted, but with little previous corroborating EEG evidence.

## Introduction

Previous recognition memory studies have used electroencephalography (EEG) to identify neural substrates of recognition memory. The ‘parietal old/new effect’ is a positive- going event-related potential (ERP) typically observed in the parietal electrodes between 500 and 800 ms and typically left lateralized. It shows greater amplitude for the correctly recognized old (hits) compared to the new (correct rejections) test items. It has been found that this effect correlates with the amount of information retrieved from the study episode (Wilding and Rugg, 1996; Curran, 2000; Wilding, 2000; Rugg and Curran, 2007; Tsivilis et al., 2015); hence, it is understood as a neural correlate of recollection. The ‘frontal old/new effect’ (or the FN400) is a frontally distributed and negative-going ERP which peaks earlier around 400 ms. The FN400 is interpreted as a neural correlate of familiarity since it shows a more negative peak for less familiar items while it typically does not vary for different amounts of recollected context information (Curran, 2000; Rugg and Curran, 2007; Tsivilis et al., 2015).

Pattern classification methods have been recently applied to EEG data to reveal novel findings during encoding of episodic memory (Jafarpour et al., 2014; Noh et al., 2014a; Anderson et al., 2015; Ratcliff et al., 2016). In Noh et al. (2014a), the classifier was used as a discriminative dimensionality reduction method to project the high-dimensional EEG data onto a discriminative space. These projections revealed neural correlates of levels of encoding in the pre- and during-stimulus periods of the study phase. This multivariate analysis directly controls for the multiple comparison problem (MCP) by effectively reducing the number of test variables. A major advantage of this approach is that it is possible to compare the brain activity across conditions even when the trial count is low, provided that a sufficient number of classifier training trials are used to establish the initial hyperplane(s) (Noh and de Sa, 2014). Hence conditions that divide subtle behavioral differences can be readily compared. In ERP studies, these data are usually ignored or combined with other conditions to acquire reasonable ERPs for analysis. This may result in losing the ability to reveal the neural mechanisms underlying subtle behavioral differences.

Our study aims to create classifiers to discriminate between the correctly identified old/new trials during the recognition phase of episodic memory experiments on a single trial basis. We also utilize pattern classifiers as multivariate analysis tools to analyze the brain activity during retrieval of recognition memory using the time domain information of the EEG data. The EEG data were collected from three separate visual memory task experiments with extrinsic source information. Two types of source information were considered in these experiments. Spatial information (the location of the item) was of interest in Experiment 1 and extrinsic color information (the color of an external frame) was of interest in Experiment 2. In Experiment 3, both source types were considered. Data collected from these experiments were used to conduct multivariate analysis via pattern classifiers. The data used were previously collected by Mollison and Curran (2012). In the experiments, subjects were asked to remember items as well as the contextual source information (side of the screen, or color of outlined box). In the test phase they were asked to indicate whether they believe they have seen the item before, and if so to give the associated source information as well as their confidence in that judgment by specifying whether they remember the source information, any other information, or whether the item is just familiar. Mollison and Curran (2012) found that even familiar judgments were associated with above chance source judgments and that the FN400 distinguished between the source-correct and source-incorrect responses only for the location-source information but not the box-color source information. In this work, we specifically train separate classifiers to extract information related to item memory (without correct source memory) and source memory (for correctly remembered items) to observe any source-dependent differences that the classifiers extract between the experiments with different source types.

The average projection values (or classifier scores) of the different source retrieval conditions and different subjective rating conditions are also compared to reveal the relationship between the different conditions and memory retrieval strength. Furthermore, data from the error conditions (incorrectly identified new trials, incorrectly rejected old trials) are projected onto the discriminative vector characterized by the different classifiers. The average projection values of these error trials are compared to those given by the other conditions and across the different projection directions.

## Materials and Methods

Electroencephalography for the current study was previously recorded in three separate visual memory task experiments (Mollison and Curran, 2012). All procedures were approved by the Institutional Review Board at the University of Colorado Boulder and were conducted in accordance with this approval. All participants gave written informed consent before the experiment.

### Experiment 1

#### Participants

The subjects were right-handed University of Colorado undergraduate students (ages 18–28, mean = 21.4) who volunteered for paid participation ($15 per hour) or course credit (17 male, 13 female). All subjects were native English speakers and had normal or corrected- to-normal vision.

#### Experimental Paradigm and EEG Acquisition

The experiment was divided into four blocks consisting of a study and recognition phase. The stimuli were color images of physical objects, animals, and people. A total of 1297 images were selected from http://www.clipart.com, the stimuli set by Brady et al. (2008), and image search on the Internet. All images were resized to 240 pixels × 240 pixels and presented on a square white background. For each subject, a total of 416 images were randomly selected as the study items (104 items per block). The test lists consisted of 100 old items from the preceding study list with 50 foil items given in random order. The first and last two stimuli in the study list were excluded from the test list to reduce primacy and recency effects.

During the study phase, the study items were presented on either the left or right side of the fixation cross. The subjects were instructed to memorize the side of the screen on which each study item was given. The spatial location of the item was considered as the source information in this experiment. A study item was shown for 1000 ms followed by an inter-stimulus interval with varying lengths (uniformly distributed within 625 ± 125 ms). A visual Gaussian noise image was shown at the locations of study item presentations whenever an item was not being presented to prevent after-image effects from the stimulus. The area containing the possible study image locations subtended a visual angle of 11.4° wide × 5.6° high.

**FIGURE 1:**
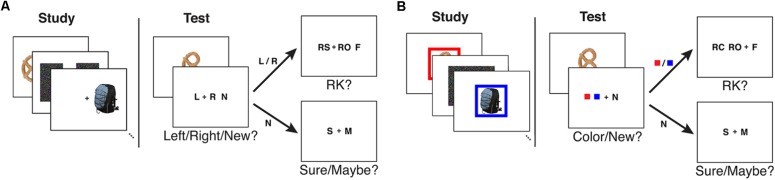
An illustration of the study and test tasks used in **(A)** Experiment 1 and **(B)** Experiment 2 as given in Mollison and Curran (2012).

In the recognition phase, a fixation cross appeared on the center of the screen for 750 ms. A test item was shown for 750 ms on top of the fixation cross followed by a 1500 ms long fixation cross. The visual angle of each test probe image was 4.3° wide × 4.3° high. Then the subjects were given two consecutive questions where the second question type depended on the subject’s answer on the first one. An inter-stimulus interval of 625 ± 125 ms followed each response. In the first question, subjects were asked to make a source/new judgment where source was the location of the item in the study phase. The first question had three options: left, right (given as L and R, respectively) and a new judgment (given as N). If the subjects responded with L or R in the first question, they were asked to give a modified R-K judgment in the second question. The R-K judgment question had three options: remember side (given as RS), remember other (given as RO), and familiar (given as F). Subjects were instructed to respond with RS if they remembered the source information, RO if they remembered something other than the source information, and F if they could not remember any details of learning the item but it looked familiar. If the subjects responded with new in the first question, they were asked to give a confidence of that response: sure (given as S) or maybe (given as M) based on how confident they were about it being a new item. See **Figure 1A** for an illustration of the study and test tasks in the experiment. The keys for left responses were assigned to the left hand (z or x key), the keys for right responses were assigned to the right hand (. or / key), and the keys for new responses were assigned to one of the outermost keys (z or / key). For the confidence judgments, the keys were set up from left to right to follow memory strength in either descending or ascending order. The familiar (F) responses and remember (RS/RO) responses were always assigned to different hands. The key assignment was fixed for a given subject, but all possible key combinations were distributed to an equal number of subjects.

**FIGURE 2:**
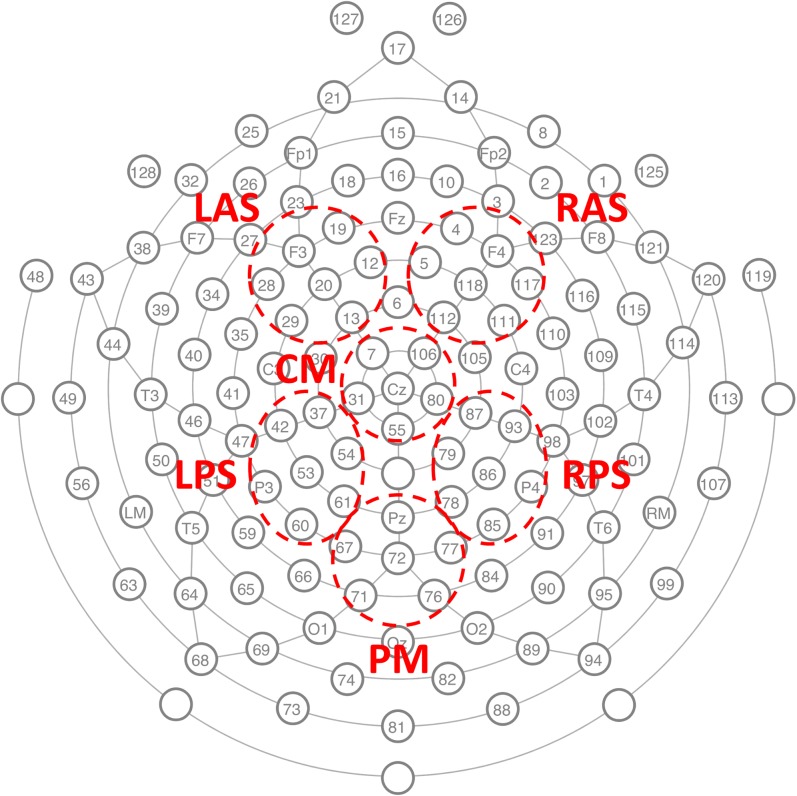
The GSN electrode locations used to record the EEG and the six channel groups on which classification analysis was conducted. LAS *left anterior superior*, RAS right anterior superior, CM *central medial*, LPS *left posterior superior*, RPS right posterior superior, and PM *posterior medial*.

EEG was recorded with a 128-channel Geodesic Sensor Net^TM^ [HydroCel GSN 200, v.2.1; Tucker (1993)] at 250 Hz sampling rate using an AC-coupled 128-channel, high-input impedance amplifier (300 MΩ, Net Amps^TM^; Electrical Geodesics Inc., Eugene, OR, United States) with a 0.1–100 Hz bandpass filter. Initial common reference was the vertex channel (Cz) and the individual electrodes were adjusted until impedance measurements were lower than 40 kΩ. **Figure 2** shows the locations of the electrodes.

### Experiment 2

#### Participants

The subjects were right-handed University of Colorado undergraduate students (ages 18–27, mean = 21.2) who volunteered for paid participation ($15 per hour) or course credit (17 male, 13 female). All subjects were native English speakers and had normal or corrected- to-normal vision.

#### Experimental Paradigm and EEG Acquisition

The stimuli set used in Experiment 1 was used in Experiment 2. In the study phase, the study items were presented with a 48-pixel wide color frame with eight possible colors (purple, green, blue, pink, red, orange, yellow, and brown). The color of the frame was considered as the source information in this experiment. Two of the four study lists used six colors and the two other study lists used the two remaining colors. Half of the subjects received the two-color condition in the even blocks and the other half of the subjects received the two-color condition in the odd blocks. All colors were randomly and evenly distributed over the study items.

During the study phase, the subjects were instructed to memorize the frame color with each of the presented study items. A study item was shown for 1500 ms followed by an inter-stimulus interval with varying lengths (625 ± 125 ms). A visual Gaussian noise image was given at the location of study item presentation whenever an item was not being presented to prevent after-image effects from the stimulus.

In the recognition phase, a fixation cross appeared for 750 ms with a preview of the two colors the subject would be choosing from immediately following the test item presentation. The number of preview colors were set to two for both six- and two-color conditions. If the test item was old (i.e., given in the preceding study list), its corresponding frame color was given in the preview. After the color preview, a test item was shown for 750 ms followed by a 1500 ms long fixation cross. Then the subjects were given two consecutive questions where the second question type depended on the subjects’ answer on the first one. In the first question, subjects were asked to make a source/new judgment where source was the frame color given with the item in the study phase. The first question had three options: two colors (given as solid color squares) and a new judgment (given as N). If the subjects responded with a color in the first question, they were asked to give a modified R-K judgment in the second question. The R-K judgment question had three options: remember color (given as RC), remember other (given as RO), and familiar (given as F). Subjects were instructed to respond with RC if they remembered the source information, RO if they remembered something other than the source information, and F if they could not remember any details of learning the item but it looked familiar. If the subjects responded with new in the first question, they were asked to give a confidence of that response: sure (given as S) or maybe (given as M) based on how confident they were about it being a new item. See **Figure 1B** for an illustration of the study and test tasks in the experiment.

EEG was recorded as for Experiment 1.

### Experiment 3

#### Participants

The subjects were right-handed University of Colorado undergraduate students (ages 18–29, mean = 20.6) who volunteered for paid participation ($15 per hour) or course credit (21 male, 17 female). All subjects were native English speakers and had normal or corrected- to-normal vision.

#### Experimental Paradigm and EEG Acquisition

The experiment was conducted in two separate sessions occurring on separate days. Each session consisted of four lists where two lists were the location source paradigm (as in Experiment 1) and two lists were the color source paradigm (as in Experiment 2). Only two frame colors (blue and yellow) were used for the color condition to match the number of location and color conditions across lists). For the first session, half of the subjects received the color condition in the even list numbers and the other half of the subjects received the color condition in the odd list numbers. The second session used the opposite order. The stimuli used in the two previous experiments were used for this experiment.

For both source conditions, a source indicator frame (color condition: blue/yellow frame, location condition: white frame on the left/right side of the screen) appeared on top of the visual Gaussian noise image prior to each study item presentation for 500 ms. Then the study item was presented inside the source indicator frame for 2000 ms followed by a slightly increased inter-stimulus interval (1125 ± 125 ms).

The timing of the recognition phase was the same as the previous experiments. However, a number of changes were made to the procedures. No color preview was given prior to test item presentation during the color condition lists. Also, the solid color squares used as source cues (as in Experiment 2) were changed to letters B and Y to better match the location conditions. Finally, both of the source responses (B and Y/L and R) were assigned to one hand and the new response (N) was assigned to the other hand. The key assignments were counterbalanced across subjects.

Electroencephalography was recorded with the same equipment as in the previous experiment except with a 500 Hz sampling rate and without the 0.1 Hz hardware high-pass filter.

### Pre-processing

Electroencephalography epochs from the recognition phase of each experiment were extracted and recalculated to average reference. In order to address any possible deficiencies in the average reference method, a subset of analyses were repeated using the reference electrode standardization technique (REST) (Yao, 2001; Dong et al., 2017; Lei and Liao, 2017), which uses reconstructed equivalent sources to re-reference electrode signals relative to a reference at infinity. The lead field for using REST had 3000 potential sources corresponding to the 128-channel HydroCel Geodesic Sensor Net^TM^ recording system used (Mollison and Curran, 2012).

Each epoch was filtered between 0.1 and 50 Hz using a 40 tap FIR filter and baseline corrected using data from −200–0 ms. Data from Experiment 3 were down-sampled to 250 Hz after the pre-processing procedure to match the sampling rate of Experiments 1 and 2.

### Classification Problem

**FIGURE 3:**
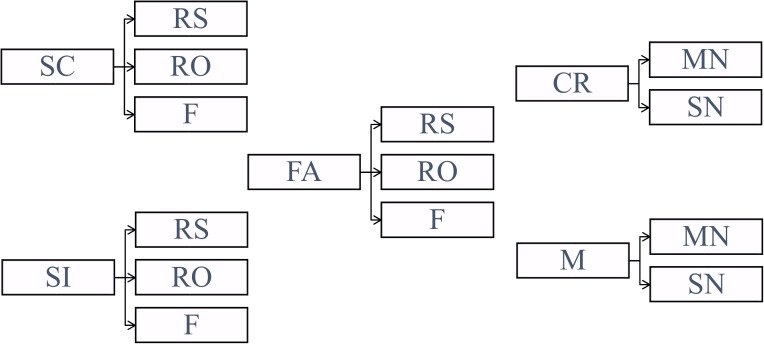
Categorization of the trials based on the subjects’ source judgments (SC, source correct; SI, source incorrect; CR, correct rejection; M, miss; FA, false alarm) and subjective ratings (RS, remember source; RO, remember other; F, familiar; MN, maybe new; SN, sure new).

Classification analysis was conducted separately on Experiment 1, Experiment 2, location source blocks from Experiment 3 (denoted as Experiment 3-location or Exp 3-loc), and color source blocks from Experiment 3 (denoted as Experiment 3-color or Exp 3-col). The data from Experiment 3 were divided into the different source conditions in order to reveal any potential differences between the location and color conditions that may correspond to ERP differences observed in Mollison and Curran (2012). Before conducting classification, the trials were divided into five conditions (*SC*: source correct, *SI*: source incorrect, *CR*: correct rejection, *M*: miss, *FA*: false alarm) based on their source judgments (1st response) as illustrated in **Figure 3**. Note that in **Figure 3** and for the rest of the paper, RS refers to remember source which includes both remember side and remember color.

The classifiers were trained to find the projection function onto the vector perpendicular to the decision boundary (we sometimes refer to these vectors as planes) which is characterized by the choice of the training conditions. The behavioral conditions corresponding to correct item retrieval (SC and SI) and correct item rejection (CR) were selected for training. As a result, three different two-class binary classifiers (SC-CR, SI-CR, and SC-SI) with probability outputs (0 ≤ *p* ≤ 1) were trained to discriminate between pairs of behavioral conditions. These probability outputs given by the classifiers are denoted as *classifier scores* in this paper. The classifiers were trained on each individual subject and only the subjects with a minimum of 25 trials for each of the 2 trained conditions (SC, SI, and CR) were included in the analysis. For each classification problem, the classifier scores were also computed for the trials which were not included in the training procedure (non-training trials).

(1) SC-CR classifier
  The SC-CR classifier (trained to discriminate between SC and CR) was expected to find the projection which maximizes the difference in the amount of information retrieved from the study episode.
(2) SI-CR classifier
  This classifier (trained to discriminate between SI and CR) was designed to discriminate between correctly retrieved old items (with incorrect source judgments) and the correctly rejected new items.
(3) SC-SI classifier
  The SC-SI classifier (trained to discriminate between SC and SI) was designed to distinguish the correctly retrieved old items with correct source judgments from those with incorrect source judgments. Hence the classifier would extract the information on source memory retrieval.

### Classification

The spatio-temporal structure of the ERPs was extracted based on previous findings on the old/new effect. Six channel groups were selected for evaluation (LAS, RAS, CM, LPS, RPS, and PM) as given in **Figure 2**. The average voltage for each channel group was computed and the data between 300 and 800 ms after test item presentation were extracted to take advantage of the ERP effects related to memory retrieval. The dimensionality of these subsequences was reduced to 5 by averaging over 100 ms length non-overlapping windows. The features from all six channel groups were concatenated to build a 30-dimensional feature vector for each trial. A binary classifier using linear discriminant analysis (LDA) with automatic shrinkage (Ledoit and Wolf, 2004; Schaefer and Strimmer, 2005) was trained to classify these feature vectors (Lotte et al., 2007; Blankertz et al., 2011). In order to avoid any overfitting to the training data, the projections for the training conditions were computed using leave-two-out (one from each class) cross-validation. In order to train with balanced classes, trials from the majority class were randomly discarded (from training) to have equal numbers of trials in each class. These trials, however, were still used for evaluation of the classifier (using a classifier trained on all the selected balanced training data). The data from the remaining conditions (e.g., Misses and False Alarms) were not used to evaluate the classifier, but were still projected onto the discriminative vector (learned from the entire balanced training set) for interpretative analysis.

### Statistical Methods

The average classifier scores (for a given classification problem) across all subjects were compared across different behavioral conditions (SC, SI, CR, M, and FA). The classifier score is a projection of the high-dimensional EEG data onto a 1-dimensional vector which is representative of the given classification problem. Paired *t*-tests were conducted on the trial-by-trial classifier scores separately for the four available datasets to compare the classifier scores of the different retrieval/subjective rating conditions. A comparison was considered to be significant only when all four separate datasets gave *p*-values below 0.05 for the conditions of interest.

It is advantageous to also visualize the EEG features utilized by the classifiers for interpreting any effects identified from the multivariate analysis using the pattern classifiers. This was done by analyzing the classifier activation patterns representing which channel, time pairs were important for classification (Haufe et al., 2014). For each source type, the 30-dimensional classifier activation pattern vector for each subject was normalized to have length 1.

In order to identify features consistent across subjects, a cluster-based method for correction for multiple comparisons was used (Maris and Oostenveld, 2007). In this method, first each spatiotemporal pixel significantly different from zero (*p* < 0.05) was identified. Then the *t*-statistic of all significant flagged neighboring pixels with the same sign was summed and the maximum absolute value over all clusters taken. This value is compared to the distribution of max absolute cluster values obtained from a permutation distribution resulting from 10,000 random permutations of class labels for each subject. Temporal neighbors were temporally adjacent time windows. Spatial groups were considered neighbors if they contained adjacent electrodes from the cap layout (see **Figure 2**). Using this rule, LAS, CM, and RAS were all mutual neighbors; CM was also neighbors with LPS and RPS; LPS and RPS were also neighbors with PM.

## Results

### Classifier Performance

Performance of the SC-CR classifier was computed based on classification of the SC and CR trials (SC-RS, SC-RO, SC-F, CR-SN, CR-MN). The significance of the performance of a classifier (whether it performs significantly over chance) was evaluated based on the number of test trials used for classification. The 95% confidence interval for the obtained accuracy was calculated using Wald intervals with small sample size adjustments (Agresti and Caffo, 2000) for each subject. Classification results were considered to be significantly over chance only when the interval did not include 50%. Results are given in **Table 1**. The overall classification accuracy for Experiment 1 (SC-CR) was 62% with 18 of 25 subjects having individual accuracies significantly over chance. When restricted to subjects with at least 50 trials in each class, the performance is somewhat better. The overall classification accuracy for Experiment 2 (SC-CR) was 59% with 17 of 28 subjects having individual accuracies significantly over chance. Experiment 3-loc (SC-CR) had an average accuracy of 57% and Experiment 3-col (SC-CR) had an average accuracy of 56%.

**Table 1A:** Classification results for Experiment 1.

Subject	SC-CR	SI-CR	SC-SI
102	0.5538	*0.4702*	*0.4693*
103	*0.4857*		
104	**0.6875**	0.5572	**0.6540**
106	**0.6720**	0.5455	0.5358
108	**0.6550**	0.4891	0.4949
109	0.5593		
110	**0.5947**	*0.4712*	*0.5*
112	*0.4953*		
113	**0.6667**	*0.328*	***0.6271***
114	**0.6746**	0.5575	**0.6442**
115	**0.5741**	0.5269	0.5517
116	0.5251	0.5183	0.4839
117	**0.5944**	*0.5148*	*0.5025*
118	**0.65**	*0.4762*	*0.5398*
119	**0.6154**	**0.5756**	0.5
120	**0.6172**	*0.5108*	***0.6090***
121	**0.5977**	0.5057	0.5356
122	0.5255	***0.6224***	*0.5420*
123	**0.6585**	*0.5603*	*0.5368*
124	*0.5649*	*0.5577*	*0.6104*
125	**0.6518**	0.5571	0.4373
126	**0.6955**	*0.4419*	***0.5981***
127	**0.6048**	*0.5530*	*0.5194*
128	**0.7474**	*0.4633*	*0.5302*
129	**0.5542**	*0.4328*	*0.4714*
Overall	0.6231	0.5090	0.5383
Overall with 50 trials/class cutoff	0.6290	0.5368	0.5397

*Overall accuracies given in the penultimate row are the accuracies over all trials from the relevant classes for subjects with 25 or more trials per class. Overall accuracies in the last row are computed over all trials from relevant classes for subjects with 50 or more trials per class. Bolded entries are significantly better than chance (p < 0.05). Results from subjects with less than 50 trials per condition are italicized.*

**Table 1B:** Classification results for Experiment 2.

Subject	SC-CR	SI-CR	SC-SI
201	*0.5113*	*0.5581*	0.5298
202	***0.6860***	*0.5915*	*0.4713*
203	*0.5444*	*0.4805*	*0.4773*
204	**0.6018**	*0.48*	***0.5787***
205	**0.5766**	0.4303	0.5126
206	0.5524	*0.5816*	*0.4563*
207	**0.6204**	*0.4177*	*0.4912*
208	**0.6349**	0.4643	**0.5701**
209	***0.6222***	*0.5714*	0.5344
210	**0.6189**	0.5356	0.5193
211	**0.6**	0.4851	0.5193
212	*0.4964*	*0.5146*	0.5723
213	**0.5942**	**0.5922**	0.5071
214	**0.6553**	0.5659	0.5211
215	**0.6728**	**0.6554**	0.5270
216	0.5475	0.5315	0.5571
217	**0.5761**	0.5484	0.52
219	0.5506	*0.4859*	*0.4757*
220	**0.6174**	**0.6171**	0.5436
221	**0.5737**	0.4826	0.5345
222	0.5504	0.4758	0.5780
223	0.55	0.4943	0.4775
224	**0.5789**	**0.5822**	**0.6053**
225	0.5538	**0.5952**	0.5431
227	**0.6757**	**0.6**	0.5410
228	*0.5533*	*0.5784*	0.5287
229	0.4588	0.4262	0.4456
230	**0.5771**	**0.5805**	0.5171
Overall	0.5904	0.5383	0.5263
Overall with 50 trials/class cutoff	0.5945	0.5416	0.5311

*Overall accuracies given in the penultimate row are the accuracies over all trials from the relevant classes for subjects with 25 or more trials per class. Overall accuracies in the last row are computed over all trials from relevant classes for subjects with 50 or more trials per class. Bolded entries are significantly better than chance (p < 0.05). Results from subjects with less than 50 trials per condition are italicized.*

**Table 1C:** Classification results for Experiment 3.

Subject	SC-CR (loc)	SC-CR (col)	SI-CR (loc)	SI-CR (col)	SC-SI (loc)	SC-SI (col)
310	**0.5665**	0.4833	*0.4538*	0.5	*0.5289*	0.5208
312	**0.6422**	**0.6257**	*0.5380*	0.5451	*0.4591*	0.5208
313	**0.57**	**0.5766**		0.5509		0.5498
315	0.5127	**0.5949**	0.5074	0.5505	0.5181	0.5064
317	**0.6039**	**0.6101**	0.5153	0.5	0.5019	0.4704
318	*0.5327*	*0.5469*	*0.6026*	*0.5185*	*0.5315*	*0.5041*
319	0.5550	*0.4706*	0.4581	*0.5399*	0.5444	0.4681
321	0.5613	**0.6291**	**0.5985**	0.5369	0.5076	**0.5647**
322	*0.5252*	*0.4818*	*0.4811*	*0.5076*	0.5170	0.4696
323	0.5615	0.5291	*0.4538*	0.5505	*0.5519*	0.5528
324	0.5244	0.5393	0.5407	0.5056	0.5430	0.5358
326	**0.6181**	**0.5700**	0.4928	0.4962	0.5516	0.4697
327	**0.6111**	0.5022		0.5586		0.5054
328	0.512	0.4971	*0.5515*	0.52	*0.5426*	0.5137
330	**0.6348**	**0.5638**	0.4462	0.4908	**0.6035**	0.4967
332	**0.5710**	**0.5698**		0.4632		0.4615
333	0.5593	0.5445		0.5724		0.5364
334	*0.5370*	0.4563	*0.5149*	**0.5984**	0.5195	0.4978
335	0.4963	**0.5714**		*0.5*		*0.4701*
336	**0.6262**	**0.6313**	*0.4545*	0.4527	***0.5685***	0.4963
337	**0.5638**	0.5481	0.5546	0.5037	0.5547	0.5
340	*0.4875*	**0.6138**	*0.3663*	0.4965	0.4785	0.5134
342	**0.5980**	0.5233	*0.4710*	0.5425	*0.4651*	0.4870
343	**0.6667**	*0.5179*	0.5652	***0.5870***	0.5059	0.4805
344	**0.5682**	0.5158	0.4930	0.4667	0.5081	0.4727
345	0.5101	0.4688	*0.5429*	0.5058	*0.5587*	0.5053
Overall	0.5736	0.5553	0.5049	0.5182	0.5261	0.5024
Overall with 50 trials/class cutoff	0.5789	0.5610	0.5153	0.5159	0.5286	0.5031

*Overall accuracies given in the penultimate row are the accuracies over all trials from the relevant classes for subjects with 25 or more trials per class. Overall accuracies in the last row are computed over all trials from relevant classes for subjects with 50 or more trials per class. Bolded entries are significantly better than chance (p < 0.05). Results from subjects with less than 50 trials per condition are italicized.*

**FIGURE 4:**
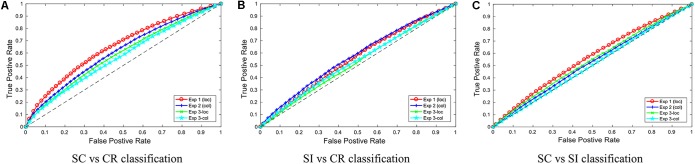
The ROC curves for the three different classification problems (**A**: SC vs. CR; **B**: SI vs. CR; and **C**: SC vs. SI) are given separately for the four individual datasets.

**Figure 4** gives the ROC (receiver operating characteristic) curves for choosing different thresholds (between 0 and 1) to make decisions between classes 1 and 2 for all 3 classification problems. **Table 2** gives the area under these ROC curves. All results were above 0.5, however, there was a variability in performance across the different classification problems. The SC-CR classifiers showed the highest performance on all four datasets. It was also found that the datasets with recordings from multiple days (Exp 3-loc and Exp 3-col) showed a slight decrease in performance compared to the single session datasets. The SC-SI classification performs better for the location source datasets relative to the color source datasets in contrast with the SI-CR classifiers.

**Table 2:** The average AUCs of the ROC curves for the three different classification problems on four experiments.

	SC-CR	SI-CR	SC-SI
Experiment 1 (loc)	0.6555	0.5586	0.5434
Experiment 2 (col)	0.6160	0.5779	0.5357
Experiment 3 (loc)	0.5916	0.5264	0.5375
Experiment 3 (col)	0.5726	0.5376	0.5108
Average	0.6089	0.5501	0.5319

*Every AUC was computed using the projections of the trials, by leave-two-out training, in the selected balanced training data*.

We redid some classifications using the reference electrode standardization technique (REST) (Yao, 2001; Dong et al., 2017). The performance of classifiers SC-CR in Experiment 1 using REST for re-referencing pre-processing showed comparative AUC (0.6571) and accuracy (0.6221) to that obtained with our usual average reference method (AUC of 0.6555 and accuracy of 0.6231). We then compared the REST method for the harder SC-SI classification in the two color source datasets, but this also resulted in no significant improvement in the classification results. Specifically for SC-SI in Exp2 (color) with REST we have AUC of 0.5206 (vs. 5357 with AR) and accuracy of 0.5191 (vs. 5263 with AR) and for SC-SI in Exp3 (color) with REST we have AUC of 0.5075 (vs. 5108) and accuracy of 0.5034 (vs. 5024).

### Analysis of the Classifier Scores

The projection weights for a given classification problem can be used to project the EEG data onto a discriminative vector. In this paper, these projection values are denoted as the classifier scores. The relationship between the average classifier scores for the different behavioral conditions represents the characteristics of the different discriminative hyperplanes (Noh and de Sa, 2014). As described in section “Statistical Methods,” the representation of the EEG data on the three different discriminative vectors were compared across the different behavioral conditions. The classifier scores were computed for each classification problem (as described in section “Classification Problem”) and the average scores corresponding to the different behavioral conditions were compared. The results were compared across the four datasets and effects with *p* < 0.05 consistently across the different datasets were considered to be meaningful (the individual comparison results are given in **Table 3**).

**Table 3:** Comparison results between the classifier scores for the SC-CR classifier.

	SC vs. CR	SC vs. SI	SC vs. M	SC vs. FA	CR vs. SI	CR vs. M	CR vs. FA	SI vs. M	SI vs. FA	M vs. FA
Exp 1 (loc)	5.79E-110	4.11E-11	6.45E-47	9.77E-08	5.22E-15	0.24363	1.08E-06	5.56E-12	0.21081	5.07E-08
Exp 2 (col)	5.53E-49	0.002464	1.93E-14	0.00092829	3.29E-27	0.14601	2.93E-07	6.62E-07	0.51457	0.0022279
Exp 3-loc	6.86E-34	0.0054622	1.36E-25	4.43E-10	3.89E-06	0.86631	0.0043021	1.34E-07	0.065969	0.0012858
Exp 3-col	3.63E-27	9.87E-05	2.25E-09	2.27E-07	7.60E-11	0.76771	0.23946	0.0001848	0.00011229	0.14554

*Paired t-tests between all possible pairs are given with their corresponding uncorrected p-values.*

**FIGURE 5:**
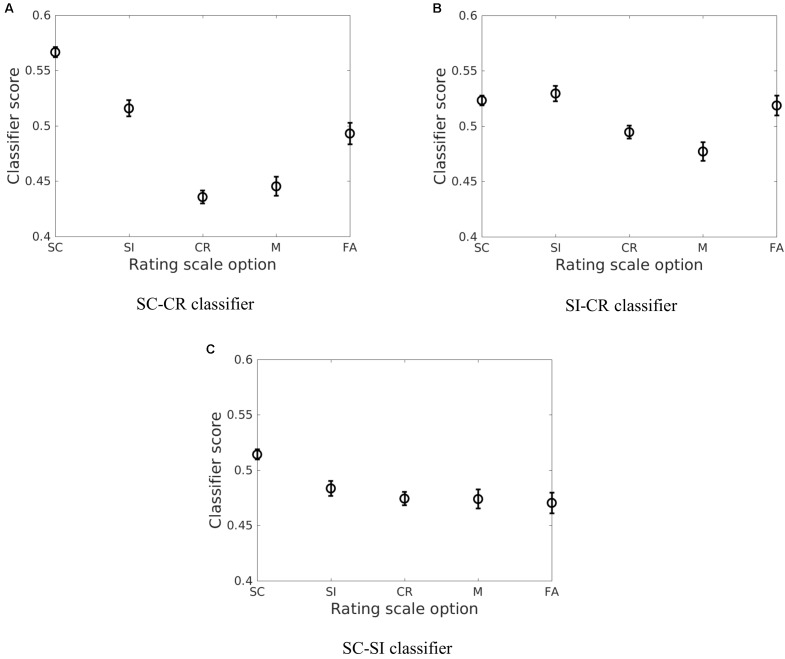
The average of the estimated means and the approximate 95% confidence intervals of the classifier scores (Hochberg and Tamhane, 1987) across the four datasets [Exp 1 (loc), Exp 2 (col), Exp 3-loc, Exp 3-col] for the five behavioral conditions (SC, SI, CR, M, and FA) for the three different classification problems (**A**: SC vs. CR; **B**: SI vs. CR; and **C**: SC vs. SI).

The correct item memory conditions (SC, SI, and CR) showed similar patterns across the different projections where SC trials gave the highest scores and the CR trials showed the lowest scores. However, the relative distance between the three conditions varied across the different discriminative vectors. It was found that the SI condition was mapped closer to the CR condition on the SC-SI plane (see **Figure 5C**) while it was mapped closer to the SC condition on the SI-CR plane (see **Figure 5B**). It was also found that the difference between the SI and CR trials were only significant (*p* < 0.05 for all four datasets) on the SC-CR and SI-CR planes (see **Figure 5**).

The relative mapping of the error conditions (M and FA) with respect to the correctly retrieved/rejected conditions (SC, SI, and CR) gave different patterns for the different projection directions. Interestingly, the source correct (SC) trials and false alarms (FA) were mapped to significantly different values on the SC-CR and SC-SI plane but not on the SI-CR plane (see **Table 4**). In contrast, the misses (M) gave values significantly lower than the two correct item retrieval conditions (SC and SI) when mapped onto the SC-CR and SI-CR plane.

**Table 4:** The uncorrected pairwise comparison results for the five behavioral conditions across the four datasets [Exp 1 (loc), Exp 2 (col), Exp 3-loc, Exp 3-col].

	SC vs. CR	SC vs. SI	SC vs. M	SC vs. FA	SI vs. CR	CR vs. M	CR vs. FA	SI vs. M	SI vs. FA	M vs. FA
SC-CR	2.35E-193	1.75E-28	6.41E-83	4.86E-26	9.18E-49	0.055638	8.80E-14	2.07E-25	0.0504	6.10E-14
SI-CR	3.46E-10	0.12096	8.04E-10	0.52851	1.44E-11	0.023804	9.54E-05	9.27E-14	0.32681	3.38E-11
SC-SI	1.52E-23	6.84E-06	8.67E-11	1.36E-10	0.0043127	0.67362	0.19108	0.0098251	0.0014469	0.32367

*The results for the different projections are given in separate rows.*

**FIGURE 6:**
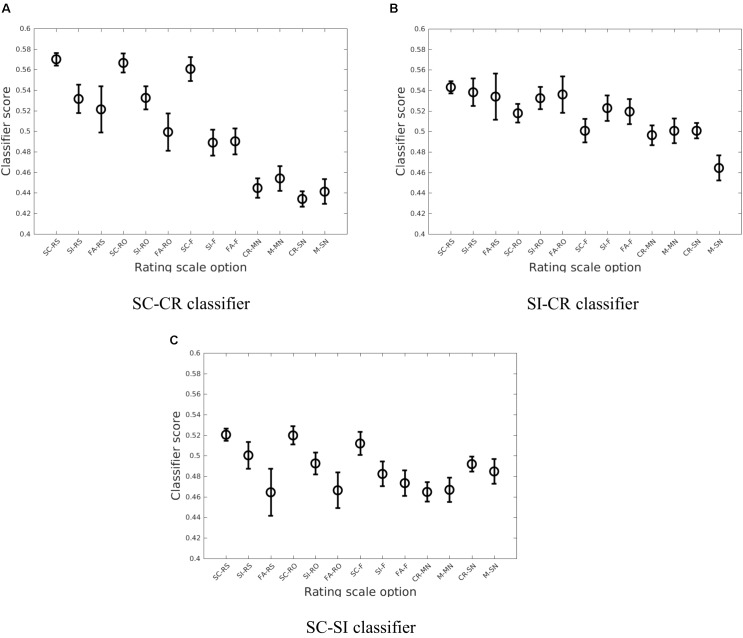
The average of the estimated means and the approximate 95% confidence intervals of the classifier scores (Hochberg and Tamhane, 1987) across the four datasets (Exp 1 (loc), Exp 2 (col), Exp 3-loc, Exp 3-col) when considering the breakdown by subjective ratings (RS, RO, F, MN, and SN) for the three different classification problems (**A**: SC vs. CR; **B**: SI vs. CR; **C**: SC vs. SI).

A similar analysis was conducted considering the different subjective ratings given to the correct item retrieval/rejection trials (SC, SI, and CR). These responses consisted of remember source (RS), remember other (RO), and familiar (F) for the SC/SI conditions and sure (SN denoting sure new) and maybe (MN denoting maybe new) for the CR condition. The error conditions (FA and M) can be similarly projected. While the classifiers generally gave a monotonic decrease in classifier scores from the RS to SN conditions, there were interesting interactions with the memory retrieval conditions as illustrated in **Figure 6**.

### Classifier Activation Patterns

**FIGURE 7:**
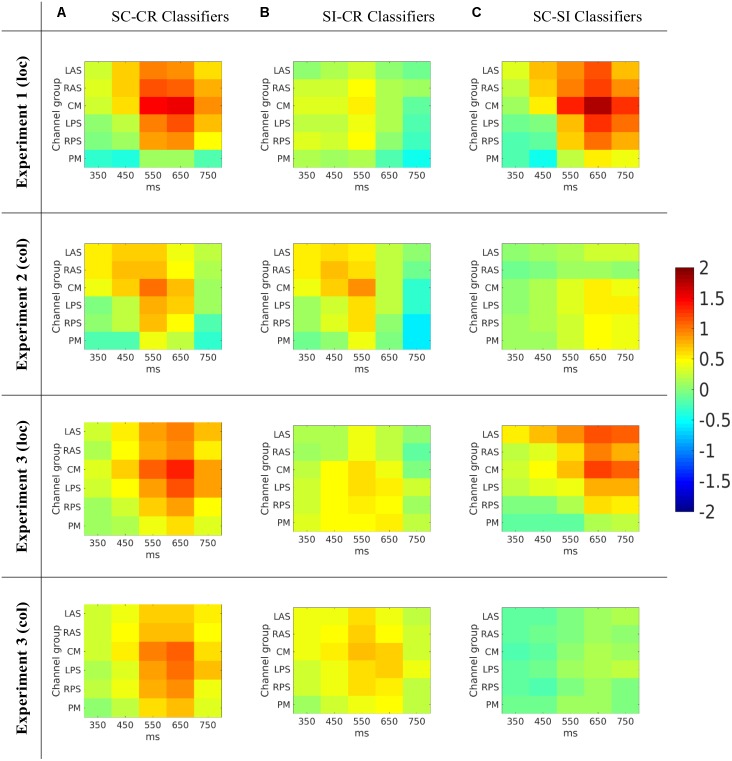
**(A)** The average activation patterns from the SC-CR classifiers averaged across all available subjects. **(B)** The average activation patterns from the SI-CR classifier averaged across all available subjects. **(C)** The average activation patterns from the SC-SI classifiers averaged across all available subjects. Note that the numbers on the *x*-axes represent the mid-point of the 100 ms window used to compute the features.

**FIGURE 8:**
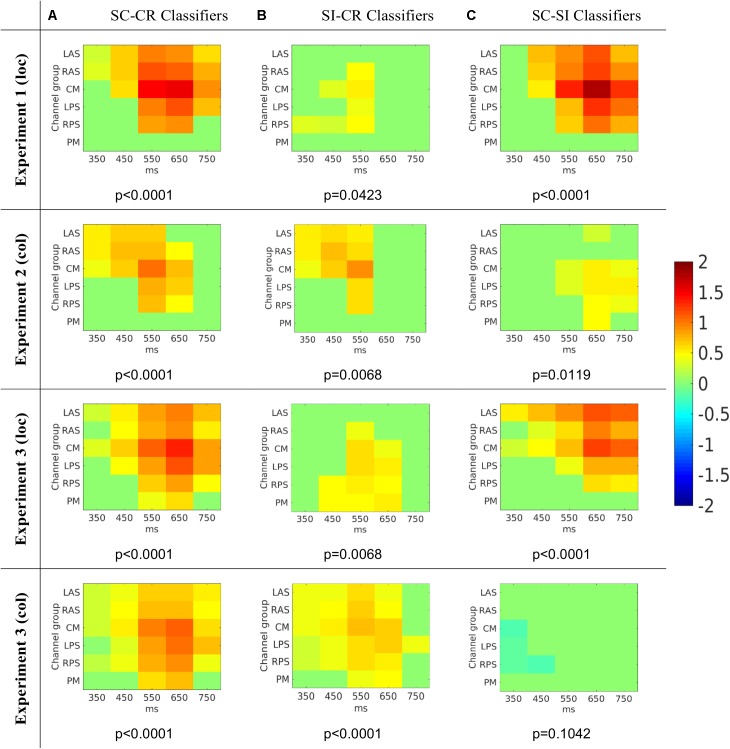
The average activation patterns from **Figure 7** but masked by the most significant cluster (with *p*-value for that cluster given below) (Maris and Oostenveld, 2007) for the three different classification problems (**A**: SC vs. CR; **B**: SI vs. CR; C: SC vs. SI). Note that the numbers on the *x*-axes represent the mid-point of the 100 ms window used to compute the features.

The activation patterns which represent the features used by the classifiers (or the characteristics of the projection weights) were compared across the three different classification problems (see **Figure 7**). The activation patterns were computed for each subject and the average activation patterns were computed by averaging the values across all four datasets. A *t*-test was conducted on each of the features to illustrate which features showed similar effects across the different subjects. Cluster based analysis (Maris and Oostenveld, 2007) was then used to control for multiple comparisons. This revealed features with values significantly above/below zero across all the subjects available for analysis. The activation patterns are given as a 2-dimensional matrix with its corresponding channel groups and time segments (the times give the center of the interval) in **Figure 7** and the most significant clusters (with significance values) are shown in **Figure 8**.

The SC-CR classifier utilized temporal features from 300 to 800 ms. The SI-CR classifier only showed consistent patterns between 300 and 700 ms and the SC-SI classifier showed consistent patterns between 400 and 800 ms for the two tasks with spatially presented contextual information (1 and 3-loc). In the two tasks with colored frames as context, there is not a strong activation pattern consistency across subjects for the SC-SI classifier. Interestingly the SC-SI (source memory) classifier has strong consistent activity across all spatial areas except PM when the source context is location. The SI-CR (item memory) classifiers have an early frontal activation when the source context is the colored outline, but a more parietal activation when the source context is the location.

The activation patterns for the three classifiers we created using the REST preprocessing (SC-CR Exp1, SC-SI Exp 2-col, SC-SI Exp 3-col) were similar to the analogous ones with average referencing.

### Classifier Scores Evolution Over Time

In the activation patterns, the characteristics of projection weights in different time intervals and channel groups were shown. The classifier scores variation across time gives a clear insight about the evolution of the separation of classes over time. To obtain the scores only under the operation with weights between 300 and 400 ms in activation patterns, the grouped EEG data after 400 ms were set to zero, and the remaining computations remained the same. In brief, the data were set to zero after the considered intervals and the trained classifier was used to get the classifier scores.

**FIGURE 9:**
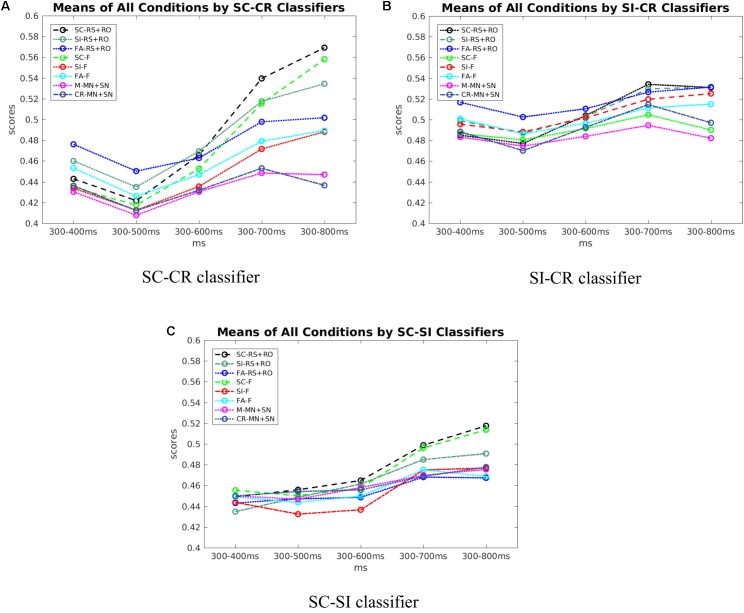
**(A)** The scores of all conditions across time by SC-CR classifiers. **(B)** The scores of all conditions across time by SI-CR classifiers. **(C)** The scores of all conditions across time by SC-SI classifiers.

**Figure 9A** shows that the scores of SC and CR trials start to be discriminable around 500–600 ms and separate further afterwards. **Figure 9B**, shows that with the SI-CR classifier, scores of SI and CR trials also start to separate around 500–600 ms. As for the SC-SI classifier in **Figure 9C**, the scores of SC trials become more separable from the scores of the SI trials after about 700 ms. Note that while the activation patterns for the SI-CR classifier show not much significant activation that is consistent between subjects after 600 ms, the classifier scores continue to separate, indicating that the activation patterns causing this separation are less consistent between subjects.

## Discussion

The results show that it is possible to predict successfully identified old vs. new items based on single-trial scalp EEG activity recorded during the retrieval episode. The prediction rate was higher for the location-source datasets and the average accuracy of the single-session datasets was higher compared to the multi-session datasets. The non-stationarity of the data between the two sessions (due to electrode position changes, impedance changes, or changes in brain-state) likely contributes to the drop in classification performance (Krauledat et al., 2007). Our analysis was restricted to time domain signals from specific channel groups known to be involved in frontal and parietal old/new effects. It is possible that accuracy could be increased by using frequency domain information from multiple electrode location and frequency bands (see for example, Hammon and de Sa, 2007; Hammon et al., 2008; Velu and de Sa, 2013; Noh et al., 2014a; Mousavi et al., 2017).

The current analysis found that the projections of the temporal information from the EEG data onto different hyperplanes show different patterns. This was evident in the relationship between the behavioral conditions of interest. We focused on the patterns which were consistent across multiple subjects and multiple datasets to compare across the different classifiers. These results suggested that the classifier may be exploiting features which are more informative for discriminating between the two behavioral conditions selected for training. It was found that the SC-SI classifier performance on the two color source datasets (Experiment 2 and the color blocks from Experiment 3) was lower compared to the location datasets (Experiment 1 and the location blocks from Experiment 3). The activation patterns for the SC-SI classifier were also not significantly consistent across subjects for the color outline source. In Mollison and Curran (2012), it was found that accurate/inaccurate judgments to the familiar responses were affected by source type where the SC trials with familiar ratings and SI trials with familiar ratings were significantly different only for the location-source datasets when comparison was conducted on a ROI centered at FCz. This suggests that the temporal information in the EEG signal may be less separable between SC and SI trials for the color datasets compared to the location datasets resulting in a lower classification performance.

The relationship between the correctly remembered conditions (where the classifier scores showed CR < SI < SC on all three discriminative vectors) suggests that these classifier scores may reflect the amount of information retrieved from the study episode. The difference in the amount of information retrieved from the study episode is maximal between conditions SC (when the correct item is retrieved from the study phase with the appropriate source information) and CR (when no information is retrieved from the study phase) which may be why the SC-CR classifier outperformed the other two classifiers. The drop in classifier performance for the SC-SI and SI-CR classifiers compared to the SC-CR classifier may be due to this innate relationship between the 3 behavioral conditions used for classifier training. The SI-CR classifier would primarily be able to utilize information related to differences between item retrieval vs. correct rejection to distinguish between the two classes. On the contrary, the SC-SI classifier would only be able to utilize information related to source memory differences between correct source retrieval vs. incorrect source retrieval in order to distinguish between the SC and SI conditions.

The activation patterns (see **Figure 8**) indicated that the classifiers used features mostly around 400 to 800 ms and gave these features higher weights. The spatiotemporal distribution of predictive features associated with the (SI - CR) classifiers (early and more frontal) were somewhat consistent with the timing and location of the FN400 only in the color-source experiments [2 and 3(col)]. Likewise the spatiotemporal distribution of predictive features associated with the (SC-SI) classifiers (later and more parietal) were somewhat consistent with the timing and location of the parietal ERP old/new effect only in the color-source experiments. In the location-source experiments, the (SC-SI) classifier had significant contributions from both early (<500 ms) and late (500–800) time periods and frontal and parietal locations. This suggests that while the SI-CR classifier may be representative of the early frontal old/new effect and the SC-SI classifier representative of the later parietal old/new effect when color is the source information, the mapping is not as appropriate when location is the source information. This is consistent with Mollison and Curran (2012) observations suggesting that familiarity contributes to source recognition for location more so than for color. The activation patterns corresponding to the SC-CR classifier took advantage of the features across all time periods (see **Figure 8**) which most likely resulted in the largest distinction between the SC and CR condition.

Additionally, the multivariate classification approach showed that trial-by-trial variation in EEG corresponding to these ERP components are predictive of subjects’ behavioral responses, which is consistent with the hypothesis that the underlying processes are influencing memory judgments. One previous study has similarly used logistic regression to predict performance on a city-size comparison task from single-trial EEG data corresponding to the FN400 (Rosburg et al., 2011). Their results showed that the relative familiarity of two cities, as indexed by single-trial FN400 measures, predicted which of the cities subjects judged as being more populous. Taken together with the current results, these classification approaches are important for establishing that EEG patterns which have been related to familiarity and recollection in ERP averages, can be shown to predict behavior on individual trials in both standard memory tasks as well as a decision making task that is influenced by memory. Overall, this strengthens the hypothesized links between these EEG patterns and behaviorally relevant memory processes.

The ERP studies of recognition memory often exclude error trials from analyses because of insufficient trials for stable ERPs in these conditions. In their original study, Mollison and Curran (2012) excluded subjects with less than 15 artifact-free trials/condition/subject and 24% of subjects would have been excluded if errors were included in the analyses. One approach for increasing the false alarm rate has been to use lures that are similar to studied items (e.g., Curran, 2000; Curran and Cleary, 2003; Nessler et al., 2001). In these cases subjects are presumed to have a high false alarm rate because similar lures are as familiar as studied items, and the familiarity-related FN400 responds similarly to hits and false alarms to similar lures. It is also common to hypothesize that false alarms to even non-similar lures are driven by familiarity. For example, the Yonelinas (1994, 1997) dual process model of ROC curves explicitly assumes that recollection does not contribute to false alarms, which are only driven by familiarity. Few ERP studies have assessed false alarms from lures that were not similar to the studied items. If familiarity differentiates “no” (CR) and “yes” (FA) responses to new items, the FN400 should be more positive to FA trials than CR trials. Although early studies that did not clearly differentiate the FN400 reported no differences between hits and false alarms (Wilding et al., 1995; Wilding and Rugg, 1996, 1997; Rubin et al., 1999), two studies that specifically focused on the FN400 did observe more positive FN400s to FA than to CR trials (Finnigan et al., 2002; Wolk et al., 2006). Wolk et al. (2006) included a very large number of test items, which resulted in an average of 105 FA trials/subject, but Finnigan et al. (2002) only averaged 12 trials/subject. The current multivariate analysis approach using pattern classifiers addresses this trial count issue by projecting the high dimensional EEG data onto a one-dimensional vector which is meaningful with respect to the experimental paradigm. The SI-CR classifier responded more strongly to FA trials than to CR, with FA being more similar to item hits (SC and SI), as would be expected if FA trials were driven by familiarity.

The relationship between the SC and FA conditions was particularly interesting. The difference between the two conditions were consistently larger across all four datasets on the SC-CR and SC-SI planes compared to the SI-CR plane as given in **Table 5** (and shown in **Figure 5**). This pattern was also evident between the SI and FA conditions, however, the distances between these two conditions were closer. Hence the representations with respect to the different classification boundaries suggest that SC and FA are more similar to each other on the SI-CR (item memory) plane compared to the other two representations. In other words, false alarms (on item information) may include information related to item retrieval while they do not include much information related to source retrieval (recollection).

**Table 5:** The difference between the average classifier scores for the SC and FA conditions are given in the top three rows.

Classifier	Exp 1 (loc)	Exp 2 (col)	Exp 3-loc	Exp 3-col	Average
**Difference between SC and FA**					
SC-CR	0.0992	0.0630	0.0647	0.0546	0.0704
SI-CR	−0.0220	0.0099	−0.0042	0.0151	−0.0003
SC-SI	0.0832	0.0456	0.0479	0.0241	0.0502

**Difference between SI and FA**					
SC-CR	0.0121	0.0353	0.0135	0.0254	0.0216
SI-CR	−0.0173	0.0258	−0.0021	0.0096	0.0040
SC-SI	0.0244	0.0171	0.0121	0.0165	0.0175

*The difference between the average classifier scores for the SI and FA conditions are given in the bottom three rows. Negative values indicate FA had larger values. The table shows that FA is mapped closer to SC and SI in the SI-CR classifier than the SC-CR and SC-SI classifiers.*

The other type of error, misses (M), were generally similar to CR in all three classifiers. Both of these conditions reflect low levels of familiarity and recollection that lead to “no” responses. Previous studies have found 300–500 ms FN400 or 500–800 ms parietal old/new differences between hits and misses, but not between CR trials and misses (Rugg et al., 1998; Curran and Hancock, 2007). Instead, Rugg et al. (1998) found differences between misses and CR were observed over posterior channels between 300 and 500 ms. The latter differences were interpreted as reflecting the activity of an implicit memory process because subjects were giving the same explicit “no” response to both old and new items, but the brain was still differentiating their memory status [although others dispute this definition of implicit memory, Voss and Paller (2008)]. Because our classifiers were trained to differentiate different levels of explicit memory, it makes sense that no major differences were observed between misses and CR in any of our results. Future work could be done to further investigate any differences by specifically involving misses in the classification training [see for example (Noh et al., 2014b)].

In summary, the present results showed that the classification analysis successfully extracts information related to retrieval strength from the EEG data. These results show that the classifier scores well represent the subjects’ behavioral performance on source retrieval (the relationship between the SC, SI, and CR conditions in **Figure 5**) and indicate that EEG item-memory and source-memory responses may be more spatially widespread than previously thought and differ between source-types. The results also indicate that retrieval strength as reflected in the classifier scores follows the subjects’ subjective ratings (**Figure 6** and **Table 6**). It was also found that the brain activity related to item memory/familiarity may be present during false item retrieval (FA trials) as well as during correct item retrieval (SC and SI trials).

**Table 6:** The uncorrected pairwise comparison results for the five subjective rating options across the four datasets [Exp 1 (loc), Exp 2 (col), Exp 3-loc, Exp 3-col].

	RS vs. RO	RS vs. F	RS vs. MN	RS vs. SN	RO vs. F	RO vs. MN	RO vs. SN	F vs. MN	F vs. SN	MN vs. SN
SC-CR	0.0031227	6.83E-27	8.57E-92	8.08E-158	5.62E-13	4.19E-66	2.31E-117	3.52E-28	1.97E-47	0.0017659

*Only the trials with correct item judgments were included in this analysis.*

## Author Contributions

TC and MM planned the EEG experiments. MM collected the data. EN, MM, TC, and VdS planned the initial analyses in this work. KL, EN, and VdS planned the temporal evolution and cluster analysis tests. EN and KL implemented the analyses. All authors were involved in drafting and editing the work and are accountable for all aspects of the work.

## Conflict of Interest Statement

The authors declare that the research was conducted in the absence of any commercial or financial relationships that could be construed as a potential conflict of interest.
